# Multifunctional Nanoparticles as High-Efficient Targeted Hypericin System for Theranostic Melanoma

**DOI:** 10.3390/polym15010179

**Published:** 2022-12-30

**Authors:** Flávia Amanda Pedroso de Morais, Ana Carolina Vieira De Oliveira, Rodolfo Bento Balbinot, Danielle Lazarin-Bidóia, Tânia Ueda-Nakamura, Sueli de Oliveira Silva, Katieli da Silva Souza Campanholi, Ranulfo Combuca da Silva Junior, Renato Sonchini Gonçalves, Wilker Caetano, Celso Vataru Nakamura

**Affiliations:** 1 Technological Innovation Laboratory in the Pharmaceuticals and Cosmetics Development, State University of Maringá, Maringá 87020-900, PR, Brazil; 2 Department of Chemistry, State University of Maringá, Maringá 87020-900, PR, Brazil; 3 Laboratory of Chemistry of Natural Products, Department of Chemistry, Center for Exact Sciences and Technology, Federal University of Maranhão, São Luís 65080-805, MA, Brazil

**Keywords:** multifunctional micelles, folate, spermine, biotin, hypericin, photodynamic therapy

## Abstract

Biotin, spermine, and folic acid were covalently linked to the F127 copolymer to obtain a new drug delivery system designed for HY-loaded PDT treatment against B_16_F_10_ cells. Chemical structures and binders quantification were performed by spectroscopy and spectrophotometric techniques (^1^NMR, HABA/Avidin reagent, fluorescamine assay). Critical micelle concentration, critical micelle temperature, size, polydispersity, and zeta potential indicate the hydrophobicity of the binders can influence the physicochemical parameters. Spermine-modified micelles showed fewer changes in their physical and chemical parameters than the F127 micelles without modification. Furthermore, zeta potential measurements suggest an increase in the physical stability of these carrier systems. The phototherapeutic potential was demonstrated using hypericin-loaded formulation against B_16_F_10_ cells, which shows that the combination of the binders on F127 copolymer micelles enhances the photosensitizer uptake and potentializes the photodynamic activity.

## 1. Introduction

Melanoma is the most aggressive and deadly skin cancer currently known. It occurs due to excessive exposure to UV-rays or genetic factors that cause changes in the melanocytes, which are responsible for producing melanin [1,2,3]. However, new therapies have been widely investigated due to their metastatic potential, recurrence rate, and high resistance to conventional treatments [2,3].

In this scenario, we highlight photodynamic therapy (PDT). This minimally invasive clinical modality uses a photosensitizer compound (PS), light, and molecular oxygen to treat cancer cells and other diseases caused by microorganisms [4,5]. After location of the PS in the therapeutic target, it is activated with the light on a specific wavelength, generating reactive oxygen species. These species are responsible for inactivating tumor cells by photochemical and photophysical mechanisms [3,6].

Hypericin (HY), a natural naphthodianthrone obtained from *Hypericum perforatum* L., also known as St. John’s wort, stands out for its promising activity against cancer cells, high singlet oxygen quantum yield (Φ∆^1^O_2_), remarkable fluorescence quantum yield (Φ_F_), and low toxicity in the absence of light [7,8]. In addition, several studies have demonstrated its PS ability and anticancer potential allied with theranostic and PDT clinical modalities [9,10,11,12].

Although HY has promising photophysical properties, its hydrophobic character promotes a low solubility in the physiological environment, which reflects its self-aggregation and reduces its phototherapeutic activity [13,14,15]. However, this problem can be solved by combining this PS with some nanomaterials that act as drug delivery systems (DDS), and promote its solubilization, stabilization, and adequate transport [14,15,16]. In general, these systems operate by passive targeting via the enhanced permeability retention (EPR effect) by solubilizing, transporting, and releasing the PS in a controlled and stable way [17,18,19,20]. This guarantees high control in the biodistribution and increased accumulation of these in the therapeutic target [21,22]. 

Pluronic^®^ amphiphilic copolymers are widely used to transport and deliver PS compounds and other drugs. Composed of (PEO-PPO-PEO) with hydrophilic groups of poly (ethylene oxide) (PEO) and hydrophobic groups of poly (propylene oxide) (PPO), they can spontaneously self-assemble in copolymeric nanomicelles [23,24,25]. This DDS contains a core formed by the hydrophobic PO groups and an outer hydrophilic layer consisting of the EO groups, the corona. The core can solubilize the hydrophobic compounds, while the corona can interact with aqueous media and/or biological fluids [23,26]. 

Several studies report the use of Pluronic^®^ as DDS as a promising strategy and advance in cancer treatment in PDT applications [21,27,28]. Recently, the conjugation of Pluronic^®^ with biomolecules, aptamers, dendrimers, antibodies, and peptides, among other ligands, has been widely reported in the literature [15,29,30]. The final effect of this combination is a noticeable enhancement of the properties of these systems as DDS. Furthermore, it can promote the active targeting of the drug, contributing to a specific interaction with the therapeutic target, which facilitates its absorption at the desired location [21,22]. Therefore, by being covalently linked on the surfaces of copolymer micelles, these ligands promote more efficiently transported drugs as well as they reduce the damage caused to healthy tissues and, consequently, the side effects of the treatment. As an example, we present the biomolecules used as ligands of the copolymer micelles addressed in this study: folic acid (FA), biotin (BT), and spermine (SN) [29,30,31,32].

FA is a water-soluble vitamin of the B complex, naturally available in its stable folate form. Its potential lies in the participation of this molecule in the processes of rapid cell proliferation, essential for the development of neoplasms, which results in the overexpression of folate receptors in cancer cells [33,34]. Therefore, the conjugation of Pluronic^®^ to FA potentiates its interaction with the tumor target, implying more excellent selectivity and efficacy of the treatment and reducing adverse effects on healthy cells [34,35,36]. 

BT, also known as vitamin B7, is another vitamin that has potential as a ligand for nanomaterials. It has specific receptors in cancer cells, such as avidin, neutravidin, and streptavidin [37,38,39]. Therefore, its conjugation with copolymers can promote good nanomaterial uptake, favoring its targeting and increasing its therapeutic efficiency [40,41,42,43]. 

Finally, natural polyamines such as SN present themselves as promising ligands for vectoring DDS. They are known as cancer biomarkers. Since neoplastic cells have deregulation of their metabolism, high production of polyamines is necessary to maintain accelerated growth [30,44,45]. On the other hand, in healthy cells, the biosynthesis of polyamines occurs at constant levels. Thus, SN can interact with cells by the polyamine transport system, which has an expressive role in cancer cells. Therefore, using these biomolecules as ligands for active targeting of copolymer systems may favor the interaction of the drug with the tumor target, as they increase the uptake, selectivity, and treatment efficacy [30,44].

In this work, the potential of active targeting the biomolecules FA, SN, and BT, conjugated to the F127 copolymer, as a DDS of the photoactive drug HY was investigated against melanoma cells. For this purpose, we conjugated these biomolecules to the F127, and obtained the materials F127-FA, F127-SN, and F127-BT. First, these biofunctionalized copolymers were used to form targeting micelles. Next, these DDS had their physicochemical properties evaluated. Then, HY was encapsulated in this DDS to form new HY formulations. Finally, the photodynamic effect of these systems was evaluated against B_16_F_10_ melanoma cells.

## 2. Materials and Methods

### 2.1. Chemical Materials

1-ethyl-3-carbodiimide hydrochloride (EDC), N-hydroxysuccinimide (NHS), succinic anhydride, 4-(dimethylamino)pyridine (DMAP), N,N-diisopropylethylamine (DIPEA), triethylamine (TEA), N,N′-dicyclohexylcarbodiimide (DCC), dimethyl sulfoxide (DMSO), 1,4-dioxane, dichloromethane, deuterium oxide (D2O), 3-(4,5-dimethylthiazol-2-yl)-2,5-diphenyltetrazolium bromide (MTT), phosphate-buffered saline (PBS), and Milex-GP syringe filter (0.22 μm, poly(ether sulfone), 33 mm) were purchased from Merck. Pluronic^®^ F127, biotin (BT), spermine (SN), folic acid (FA), HABA/Avidin reagent, fluorescamine, diphenylhexatriene (DPH), and pyrene were purchased from Sigma-Aldrich. RPMI-1640 medium, L-glutamine, and fetal bovine serum (FBS) were obtained from Invitrogen. HY was synthesized as previously reported [46].

### 2.2. Instruments

Spectrophotometric analysis was performed using UV-Vis (Cary-60 Spectrophotometer, Varian Technologies) or on a plate spectrophotometer (PowerWave XS-BioTek, at 570 nm). Fluorescence analysis was performed using a spectrofluorometer coupled to a temperature control Peltier (Cary Eclipse Spectrofluorometer, Varian-Agilent Technologies, São Paulo, Brazil). Nuclear magnetic resonance (NMR) spectra were recorded using a Bruker AVANCE III HD spectrometer (300 MHz). The Fourier transform spectrometer was equipped with a direct field gradient and probe of 5 mm. Chemical shifts (δ) were given in ppm using residual internal HDO (δ 4.70) as reference. All spectra were processed with Bruker TopSpin 3.1 software. Size, polydispersity index (PDI), and zeta potential were evaluated using dynamic light scattering (DLS) on a Nanoplus Zeta/Nano Particle Analyzer, Gerbrunn, DE. 

### 2.3. Synthesis of BT-Conjugated F127 Pluronic^®^ (F127-BT) 

BT (0.50 g, 2.00 mmol), EDC (0.33 g, 2.10 mmol), and NHS (0.24 g, 2.10 mmol) were dissolved in anhydrous dichloromethane (20 mL). The solution was maintained under stirring at room temperature in an argonium atmosphere for 1 h. F127 copolymer (1.00 g, 0.08 mmol) dissolved in DMF (30 mL) was added dropwise with a dropping funnel under dried argon over 1 h. The reaction mixture was carried out under vigorous stirring for an additional 48 h at room temperature. Unreacted BT was extracted using 10% NaHCO_3_ solution. Then, the organic phase was frozen overnight and filtered. The solvent was removed under reduced pressure and the F127-BT copolymer was dried overnight under vacuum. The crude product was submitted to dialysis (MWCO 3.5 kDa) against ultrapure water over 72 h (3 × 500 mL). The resulting solution was lyophilized to yield 0.84 g (84%) of F127-BT as a white solid. ^1^H NMR confirmed the product, and the percentage of BT was determined by HABA/Avidin assay [31,47]. 

### 2.4. HABA/Avidin Assay

The amount of available BT on F127-BT was determined by HABA/avidin assay [31,47]. First, the powdered HABA/avidin reagent was reconstituted in 10 mL of deionized water. Then, 900 µL of HABA/avidin solution was poured into 1 mL of deionized water. Finally, 100 µL of F127-BT was added. The absorbance was recorded before (A_500_^HABA/Avidin^) and after (A_500_^HABA/Avidin+F127-BT^) this addition. The amount of the available BT was calculated using Equation (1):

(1) $$\frac{\mu \mathrm{mol}\;\mathrm{BT}}{m}=\frac{A_{500}^{\mathrm{HABA}/\mathrm{Avidin}}-A_{500}^{\mathrm{HABA}/\mathrm{Avidin}\;+F127-\mathrm{BT}\;}}{34})\;\times \;\left(10\right)$$

34 = mM extinction coefficient at 500 nm and 10 = dilution factor of sample into cuvette [48].

### 2.5. Synthesis of SN-Conjugated F127 Pluronic^®^ (F127-SN)

F127 (1.00 g, 0.08 mmol of OH groups), succinic anhydride (0.8 mg, 0.08 mmol), DMAP (270 mg, 0.08 mmol), and TEA (9.8 mg, 0.08 mmol) were dissolved in 1,4-dioxane (10 mL). This mixture was maintained under vigorous stirring in the dark at room temperature for 48 h under dried argon. Then, the solvent was removed by rotary evaporation, and the product was solubilized in distilled water (10 mL). The crude product was submitted to dialysis against ultrapure water over 72 h (3 × 500 mL). The resulting solution was lyophilized to yield 0.78 g (78%) of F127-COOH as a white solid. The percentage of carboxylation was determined by titration with KOH (normalized from titration with potassium hydrogen phthalate) and bromothymol blue as indicator.

For a solution of F127-COOH (0.5 g, 0.04 mmol) in DCM (30 mL), under argonium atmosphere, EDC (12.4 mg, 0.08 mmol) and NHS (9.2 mg, 0.08 mmol) were added and the reaction mixture was stirred at room temperature for 1 h. After that, a solution of SN (10 mg, 0.08 mmol) in DCM (30 mL) was added by syringe pump over 2 h, and the mixture was allowed to stir at room temperature by 48 h. The solvent was eliminated under reduced pressure, and the resulting product was maintained under vacuum for 24 h. The crude product was diluted in 10 mL of ultrapure water and purified by dialysis against ultrapure water. The resulting solution was lyophilized to yield 0.412 g (84%) F127-SN as a colorless solid. The confirmation of the product was estimated at ^1^H NMR and the percentage of SN was assessed by fluorescamine assay [49].

### 2.6. Fluorescamine Assay

The kinetic profile of the interaction of fluorescamine (0.3 g/L) with SN (76 mg/L) was previously evaluated by fluorescence emission spectra (λ_exc_ = 390 nm, λ_max_ = 486 nm, slits 5/5, optical path of 1.00 cm, pH = 9).

Subsequently, several samples were prepared in the same condition as the kinetic evaluation but with different increasing concentrations of SN (0–76 mg/L). These samples were vortexed for 15 s at 1000 rpm and stored for the period estimated by the kinetics. Then, the fluorescence emission spectra were collected to obtain the curve (λ_exc_ = 390 nm, slits 5 and 5, optical path of 1.00 cm at 37.0 °C).

### 2.7. Synthesis of FA-Conjugated F127 Pluronic^®^ (F127-AF)

FA (54.6 mg, 0.12 mmol), EDC (18.6 mg, 0.12 mmol), and NHS (13.8 mg, 0.12 mmol) were dissolved in anhydrous dichloromethane (30 mL). The mixture was maintained under vigorous stirring at room temperature for 1 h under dried argon. F127 (0.5 g, 0.04 mmol) in DMSO (20 mL) was added dropwise, and the mixture was allowed to react at room temperature for 48 h. The unreacted FA was neutralized with sodium bicarbonate. The crude product was submitted to dialysis against ultrapure water over 72 h (3 × 500 mL). The resulting solution was lyophilized to yield 0.32 g (65%) of F127-FA as a yellow solid. The product and the FA percentage were confirmed at ^1^H NMR and by calibration curve, respectively.

### 2.8. Folate Assay 

**Figure 1:**
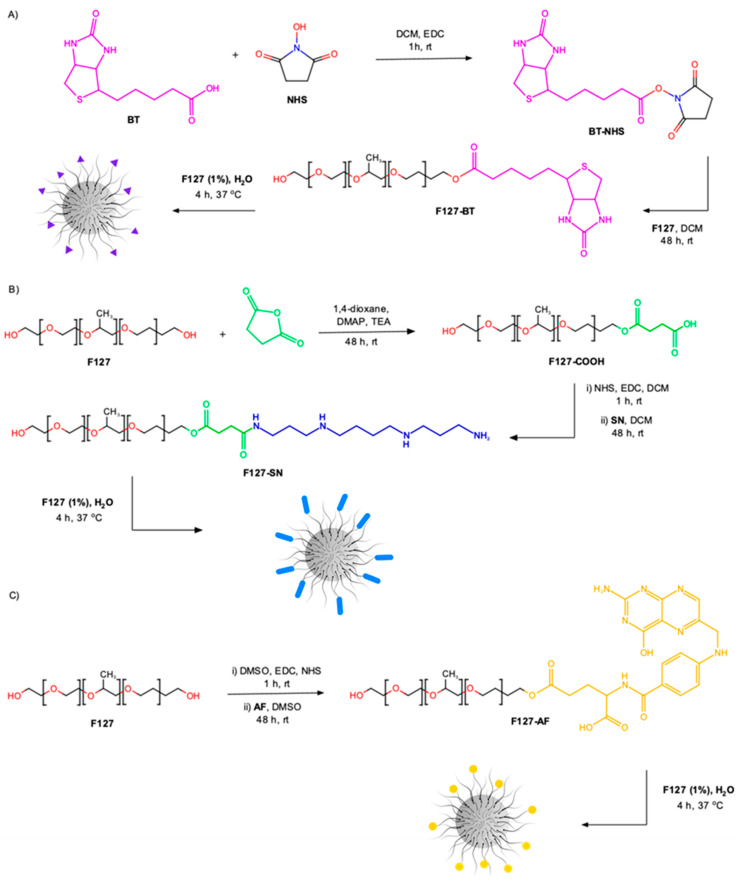
Schematic representation of the reaction and micelle preparation to obtain: (**A**) F127-BT, (**B**) F127-SN, and (**C**) F127-AF.

**Figure 2:**
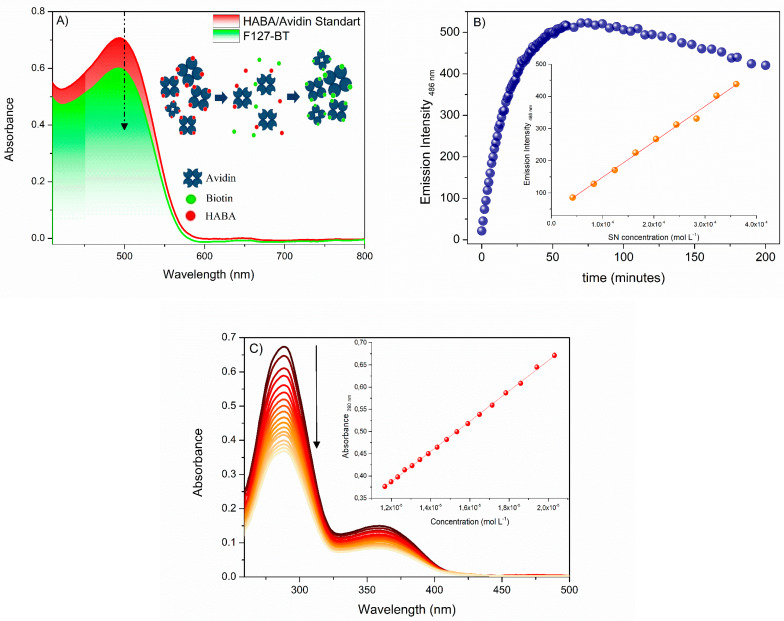
(**A**) HABA/Avidin assay for the determination of BT bound to the F127. Schematic insert of HABA/Avidin behavior in the presence of biotin. (**B**) Kinetic profile of fluorescamine with spermine addition. Insert calibration curve obtained to determine the percentage of SN bound to the F127. (**C**) UV-Vis electronic absorption spectra and curve obtained for the determination of the percentage of AF bound to F127 (30.0 °C, λ_max_ = 290 nm). The black arrow on the side indicates the direction of the fluorescence signal with the titrations.

The characterization of the FA groups in the F127 copolymer was performed by UV-Vis. In the UV-Vis absorption spectrum, the absorption band at 286 nm was due to the chromophore group and the *p*-aminobenzoic acid moiety in the chemical structure of F127-FA (Figure 1). The degree of FA conjugation onto the F127 copolymer was determined to be about 11% from the calibration UV-Vis curve (Figure 2).

### 2.9. Critical Micelle Concentration (CMC)

CMC was determined at 30.0 °C for all modified copolymers by fluorescence measurement using pyrene as a probe [50,51]. A known weight of this compound was added to ethanol and sonicated until homogeneity. The experimental 5 µmol/L of pyrene was prepared from the stock by dilution wherein the ethanol concentration was inferior to 0.2% (*v*/*v*). This solution was diluted by titration with the modified copolymers (0.01–7% *w*/*v*). The ratio of the intensity of I_1_ (373 nm) and I_3_ (383 nm) of pyrene allowed the CMC determination (λ_exc_ = 330 nm) [52]. The F127 without modification was used as standard.

### 2.10. Micelle Preparation

The modified copolymers (1.0%, *w/v*) were dissolved in ethanol. The solvent was rotary evaporated at 40 °C and the resulting solid matrix was dried under vacuum overnight. This matrix was hydrated with 5.0 mL of Milli-Q water. It was shaken at a rate of 50 cycles per minute at 37 °C for additional 4 h. Particle size, zeta potential, and polydispersity were determined only for the modified copolymers as micelles in a Nanoplus Zeta/Nano Particle Analyzer instrument (37.0 °C, n = 5 ± SD).

### 2.11. Critical Micelle Temperature (CMT)

CMT was determined for all modified copolymers by absorption measurement using DPH as a probe [53,54]. The copolymeric micelles (1.0% *w/v*) were obtained as previously described with one step of modification, the addition of 7 µmol/L of DPH. The absorption spectra of DPH were collected in the 300–500 nm range after 20 min of thermal stabilization at 15.0–50.0 °C and allowed the CMT determination (λ_max_ = 356 nm) [53,54,55,56,57]. The F127 without modification was used as standard.

### 2.12. MTT Assay

In vitro cytotoxicity was performed by MTT with mouse melanoma cell line B_16_F_10_ (ATCC CRL-6475). The cell culture RPMI-1640 was supplemented with L-glutamine, heat-inactivated fetal bovine serum (FBS, 10%), and incubated at 37 °C in a CO_2_ atmosphere (5%). The cells were seeded in 96-well microplates containing RPMI-1640 medium at 2.5 × 10^5^ cells/mL per well and maintained at 37 °C and CO_2_ (5%) for 24 h. All copolymeric formulations were added at increasing concentrations of HY photosensitizer compound (0.078–5 µmol L^−1^) which was added during the solid matrix obtention process. 

The photodynamic treatment was performed for 40 min using a visible light source (550−625 nm, 35 mW cm^−2^, 46.8 J cm^−2^ of light dose). After treatment, the cells were incubated for 48 h at 37 °C and CO_2_ (5%). Then, cells were washed with PBS (pH 7.2) and incubated with MTT (2 mg/mL) for 4 h. The unreacted dye was removed, and the black-blue formazan crystals were dissolved in 150 µL of DMSO in each well. The absorbance reading was taken at 570 nm on a plate spectrophotometer (PowerWave XS-BioTek). The percentage of viable cells was calculated concerning the control, and the cytotoxic concentration was determined by logarithmic regression analysis. All experiments were performed in triplicates (n = 3 ± SD).

## 3. Results and Discussions

### 3.1. Synthesis

F127-BT copolymer was prepared in one-pot synthesis employing EDC and NHS to activate the carboxylic group of BT for direct conjugation with the hydroxyl ending group of F127 copolymer via esterification reaction (Figure 1). The purification of F127-BT was performed by dialysis membrane against ultrapure water. The synthesis of F127-SN was carried out in a two-step process (Figure 1B). First, the F127 copolymer was subjected to activation by inserting carboxyl terminal groups via a ring-opening reaction using succinic anhydride and DMAP. In the next step, F127-COOH (containing 87% of -COOH end groups) was submitted to an amidation reaction by using EDC and NHS. The resulting *N*-hydroxysuccinimidyl ester reacted with the primary amine group of SN to ratify F127-SN (Figure 1B).

The FA-conjugated F127 was conducted by direct esterification reaction between -OH of F127 and carboxyl end groups in FA using EDC and NHS (Figure 1C). Both F127-SN and F127-FA were purified by dialysis membranes against ultrapure water

### 3.2. ^1^H NMR Characterization

The ^1^H NMR technique was performed to elucidate the functional groups sequences of BT-, SN-, and FA-conjugated F127 copolymers. In the spectrum of F127-BT (Figure S1), the highest intensity signals resonated at δ 1.1, 3.5, and 3.6 and were ascribed, respectively, to methyl, methynic, and methylene hydrogens (H-1, H-8, and H-9), which correspond to the repetitive units the EO and PO. The signal resonating at δ 3.9 (H-10) was attributed to methylene hydrogens directly connected to ester moiety, which supported that the esterification reaction was performed successfully. The signals group marked as H-2, H-3, H-4, and H-5 resonated at δ 1.5–2.3 and were assigned to methylene hydrogens of the aliphatic chain moiety of the biotin molecule. The hydrogens signals correlated to ureido moiety H-6, H-6’, and H-7 were observed at δ 2.7 and 2.9, respectively, while the signals resonated at δ 4.2 and 4.5 and were marked, respectively, as H-11 and H-12 and were attributed to hydrogens of tetrahydrothiophene moiety. Additionally, the δ value (2.3) observed in H-5 was in agreement with the typical δ value α-hydrogens of the ester group, confirming the covalent conjugation of biotin with F127 copolymer

In the spectrum of F127-COOH, four high-intensity signals were observed, which were related to hydrogens of the repeating units EO and PO of the copolymer (Figure S2).

The signal resonating at δ 3.6 was assigned to hydrogens of EO (H-2). The signals resonating at δ 3.4 and 1.0 were assigned to the hydrogens H-3 and H-5 of the PO moieties. Compared to the F127 spectrum (Figure S3), two new signals were revealed in the spectrum of F127-COOH. The signal resonating at δ 4.2 was ascribed to methylene hydrogens of the EO unit directly connected to the succinate group (H-1). On the other hand, the signal resonating at δ 2.5 was assigned to α- and β-hydrogens of the carboxyl group (H-4), as a confirmation that the F127 activation was performed successfully.

The spectrum of copolymer F127-SN revealed five additional signals related to the insertion of SN on F127-COOH (Figure S4). The signal resonating at δ 3.6 was assigned to hydrogens of EO (H-2). The signals resonating at δ 3.5 and 1.1 were assigned to the hydrogens H-3 and H-10 of the PO moieties. The signal resonating at δ 4.2 was ascribed to methylene hydrogens of EO unit directly connected to the succinate group (H-1). The signals corresponding to α-methylene hydrogens of amine groups (H-4 and H-5) were observed at δ 2.8 and 2.7, respectively, while the signals resonating at δ 2.1 and 1.9 were ascribed to β-methylene hydrogens of the amine group (H-8 and H-9). The α- and β-hydrogens of the carboxyl group were observed at δ 2.5 and 2.4 (H-6 and H-7), respectively. 

In the spectrum of F127-AF, (Figure S5), the signals resonating at δ 3.5, 3.3, and 1.0 were assigned to EO and PO units. The signals resonating at 8.6 and 7.7 were assigned to pteridine (H-1) and amidine (H-2) hydrogens. The signals relative to aromatic hydrogens H-3 and H-5 were observed at 7.6 and 6.6, respectively. The signal at 6.8 was ascribed to amine hydrogen (H-4). The signals relative to H-6 and H-7 were observed at δ 4.4 and 4.2, respectively. Finally, the signals that resonated at δ 2.1 and 1.9 were related to α- and β-hydrogens of the ester group and assigned as H-10 and H-11. The high chemical shift values observed for the hydrogen H-2, H-7, H-10, and H-11 compared to those observed in the FA spectrum H-h, H-c, H-b, and H-a (Figure S6) support that the FA binding to F127 copolymer was performed successfully. Additionally, the pattern of signals observed in the spectrum of F127-FA agreed with previous reports relative to F127-FA [54,57].

### 3.3. Percentage of FA, BT, and SN on F127

The percentage of FA, BT, and SN ligands was performed using a calibration curve, HABA/Avidin, and fluorescamine assay, respectively (Figure 2).

HABA/Avidin assay is a suitable reagent for determining biotin in the F127-BT synthetic product. This reagent can form a complex with intense absorption bands in the visible region and a known molar absorptivity coefficient at 500 nm [47]. After adding biotinylated compounds to the solution, HABA is replaced by the biotin of F127-BT, forming a new complex F127-BT/Avidin [31]. As a result, a decrease in absorption was observed. Figure 2A shows the UV-Vis spectra before and after adding F127-BT to the HABA/Avidin solution. The spectral variation of absorbance allowed the quantification of biotin bound to F127-BT using Equation (1). The available amount of biotin in F127-BT micelles was 0.0379 µmol mL^−1^, which corresponds to approximately 13% of BT bound to the F127-BT synthetic product. Similar results were observed for biotin-conjugated to poly(ethylene glycol)-b-poly(azidoethyl methacrylate)-bpoly(methyl methacrylate) (PEG-b-PAzEMA-b-PMMA) triblock micelles and for biotin-targeted Pluronic^®^ P123/F127 mixed micelles (both 10 % of yield [31,40]). These results can be attributed to the biotin-conjugating experimental conditions as well as to the rigidity of the micelles interfaces, which can prevent avidin molecules from binding to the biotin and forming the complex.

The percentage of SN binding in the F127 was determined using the fluorescamine assay [49,55]. Fluorescamine is a nonfluorescent compound that reacts with primary amines forming a fluorescent species. This species allows the detection of the presence and the quantitative determination of primary amines [55,56]. Therefore, this technique is well known for being selective and sensitive to the amine content in the sample, thus, the synthetic product. As shown in Figure 2B, it was necessary to evaluate the kinetic period before obtaining the calibration curve. After that, the measure of SN binding to F127 was determined, showing an ending group amine conversion of 32% for F127, a value very similar to those reported in the literature for synthesis involving polyamines [49,55,56]. Finally, the quantification of FA conjugated to the copolymer F127 was measured using a calibration curve performed with FA in DMSO (11% of yield). The literature reports some studies involving this synthesis which obtains the same product, thus, F127-FA. However, most of those do not quantify the FA content in F127-FA copolymer due to the low yield, which is generally close to 5% [29,57].

### 3.4. Micelle Characterization

In aqueous solution and under specific conditions, amphiphilic triblock copolymers such as Pluronic^®^ can self-assemble into highly organized structures as micelles. However, the concentration range and the temperature are characteristic parameters and, therefore, variable in the face of material modifications. Before that, the critical micellar concentration (CMC) and the critical micelle temperature (CMT) of the modified materials were evaluated using the copolymer without its functionalization as a standard. Experimental values are shown in Table 1.

Determination of the CMC of the modified materials was performed using pyrene as a fluorescent probe. In the absence of micelles, that is below the CMC, the pyrene molecules can determine the hydrophilic environment by the high ratio of their vibronic bands I_1_ and I_3_. Similarly, with the addition of copolymers to this system, the configuration and arrangement of the pyrene molecules are affected. Thus, in and above the CMC, the pyrene molecules change their microenvironment in the presence of micelles [58,59]. The high hydrophobicity of pyrene allows it to be inside the micelles, and consequently, the ratio between bands I_1_ and I_3_ decreases. Therefore, the plot I_1_/I_3_ versus F127 concentration enables the CMC determination of the systems. The experimental values suggest that the material modified with FA was the one that suffered the most changes in its CMC value when compared to its analogs and the standard F127 (without modification), which increased the CMC value more than 7-fold. These changes probably occur due to the increased hydrophilicity attributed to the folic acid molecules that alter the medium to be more hydrated, increasing the CMC of the biofunctionalized copolymer. The biofunctionalized system with biotin presented similar results, showing an increase of about 4-fold in CMC. On the other hand, despite being the material with the highest percentage of modification, the copolymer functionalized with SN was the one that showed a minor change in its CMC compared to F127 without modification. Thus, one factor that generates the influence of the CMC is the hydrophilicity of the ligand. Furthermore, the F127-SN was the only system that suffered a drop in this value, probably associated with its hydrophobicity. In fact, according to Table 1, the F127-SN system showed fewer physical-chemical changes in all parameters evaluated. Another example is the CMT experimental results using the DPH compound as a probe (Table 1). Similar to pyrene, the band at 356 nm is known to highlight changes in the DPH microenvironment. The spectral profile as a function of temperature allows the variation suffered by this compound due to the formation of the micelles. The experimental values confirm that the F127-SN was the product that presented the slightest variations concerning the F127 without modification (less than 3 degrees), while its analogs showed 5.5 and 4.7 (F127-AF and F127-BT, respectively). As a number of distributions, particle size values confirm the formation of the micelles (Dh = 13–20 nm). Subtle differences in micelle Dh can be associated with the bioligands, which do not significantly alter this parameter and the PDI. The exception occurs for micelles containing SN, which showed higher Dh and PDI values. In addition, it is possible to observe a significant decrease in the zeta potential for all modified micelles compared to their standard micelles without synthetic modification. These zeta results can be correlated to an improvement in the physical stability of these systems. Again, the F127-SN micelles showed less oscillation to the pattern, suggesting that the physicochemical properties of this new material slightly differ from F127 micelles.

### 3.5. MTT Assay

After determining the physicochemical parameters of these new materials, they were used to carry the photoactive drug HY in photodynamic assays against B_16_F_10_ cells. The experiments were carried out in increasing concentration of HY 0.039 μmol L^−1^ to 5 μmol L^−1^ in the presence or absence of illumination (Table 2).

Table 2 shows the photodynamic activity of the HY formulations in the modified micelles in the presence and absence of light. The variation in the CC_50_ values confirms the target of the modified HY formulations. HY-loaded F127 micelles without modification present CC_50_ values at least 1.6-fold higher than the other modified systems. These results strongly suggest these new HY formulations can improve the photodynamic inactivation of the B_16_F_10_ cells. Furthermore, combined with the physicochemical parameters, there is an indication of the enhanced permeability and retention (EPR) effect, pointing to the potential of these systems developed for cancer treatment using this PDT clinical modality. Furthermore, no variation was observed when the cells were exposed to the modified micelles; thus, the carrier itself does not cause any damage to the cell lines, even in the presence of illumination (not shown). The same result was observed for the formulations containing HY but in the absence of light, which indicates all HY formulations do not present toxicity in the dark.

## 4. Conclusions

In this work, biotin (BT), spermine (SN), and folic acid (FA) were covalently linked to F127 copolymer to obtain a new drug delivery system designed for HY-loaded PDT treatment against B_16_F_10_ cells. ^1^H NMR technique was used to confirm the functional groups sequences of materials and the quantification of the binders, which were performed by HABA/Avidin reagent, fluorescamine assay, and calibration curve. The modified copolymers were characterized by CMC, CMT, DLS, PDI, and zeta potential. The results indicate the hydrophobicity of the binders can influence the physicochemical parameters. Furthermore, the phototherapeutic potential of F127 multifunctional micelles loading HY was demonstrated by in vitro assay using B_16_F_10_ cells. The findings showed that the combination of the binders on F127 copolymer micelles is the main factor in enhancing the HY uptake by B_16_F_10_ and can potentialize photodynamic activity.

## Figures and Tables

**Table 1:** Micelles characterization: critical micellar concentration (CMC), critical micellar temperature (CMT), hydrodynamic diameter (Dh), polydispersity (PDI), and zeta potential (ζ). Dh, PDI, and ζ were performed at 37.0 °C.

Sample	CMC (mol L^−1^)	CMT (°C)	Dh (nm)	PDI	ζ (mV)
F127-SN	5.2 × 10^−5^	23.4	19.9 ± 1.9	0.37 ± 0.12	−7.2 ± 0.2
F127-BT	3.6 × 10^−4^	21.2	13.2 ± 0.5	0.15 ± 0.01	−14.9 ± 0.9
F127-AF	6.6 × 10^−4^	20.4	15.7 ± 0.9	0.27 ± 0.02	−13.7 ± 1.1
CMC_F127_ = 9.02 × 10^−5^ mol/L Dh_F127_ = 14.6 ± 0.53 at 37.0 °C CMT_F127_ = 25.9 °C ζ _F127_ = −2.32 ± 0.58 at 37.0 °C					

**Table 2:** Results obtained after photodynamic treatment for formulations with and without HY. The formulations without HY were also used as a control and showed no cytotoxic effect (CC_50_ > 5 µmol L^−1^), even in light.

Formulations without HY	B_16_F_10_ Cells CC_50_ (µmol L^−1^) with Illumination	B_16_F_10_ Cells CC_50_ (µmol L^−1^) without Illumination
**F127**	>5	>5
**F127-SN**	>5	>5
**F127-FA**	>5	>5
**F127-BT**	>5	>5
**Formulations** **with HY**	**B_16_F_10_ cells** **CC_50_ (µmol L^−1^)** **with illumination**	**B_16_F_10_ cells** **CC_50_ (µmol L^−1^)** **without illumination**
**F127**	1.28 ± 0.11	>5
**F127-SN**	0.77 ± 0.03	>5
**F127-FA**	0.47 ± 0.02	>5
**F127-BT**	0.24 ± 0.02	>5
CC_50-_cytotoxic concentration for 50%		

## Data Availability

Not applicable.

## Supplementary Materials

The following supporting information can be downloaded at: https://www.mdpi.com/article/10.3390/polym15010179/s1, Figure S1: 1H NMR spectra of F127-BT copolymer in D2O at 25 °C and 300 MHz. Figure S2: 1H NMR spectra of F127-COOH copolymer in D2O at 25 °C and 300 MHz. Figure S3: 1H NMR spectra of F127 copolymer in D2O at 25 °C and 300 MHz. Figure S4: 1H NMR spectra of F127-SN copolymer in D2O at 25 °C and 300 MHz. Figure S5: 1H NMR spectrum of FA acquired in DMSO-d5 at 300 MHZ and 25 °C. Figure S6: 1H NMR spectra of F127-FA copolymer in D2O at 25 °C and 300 MHz.
