# Supplementary material for: Multifunctional Nanoparticles as High-Efficient Targeted Hypericin System for Theranostic Melanoma
Source: Polymers (Basel). 2022 Dec 30;15(1):179. doi: 10.3390/polym15010179 (PMC9824163; doi:10.3390/polym15010179)

Supporting Information

Multifunctional nanoparticles as high-efficient targeted hypericin system for theranostic melanoma

Flávia Amanda Pedroso de Morais [1,2*], Ana Carolina Vieira de Oliveira[2], Rodolfo Bento Balbinot [1], Danielle Lazarin-Bidóia [1], Tânia Ueda-Nakamura [1], Sueli de Oliveira Silva [1], Katieli da Silva Souza Campanholi [2], Ranulfo Combuca da Silva Junior [2]; Renato Sonchini Gonçalves [3]; Wilker Caetano [2]; Celso Vataru Nakamura [1*].

[1] *Technological Innovation Laboratory in the Pharmaceuticals and Cosmetics Development, State University of Maringá, 87020-900 Maringá - PR, Brazil.*

[2] *Department of Chemistry, State University of Maringá, 87020-900 Maringá - PR, Brazil.*

[3] *Laboratory of Chemistry of Natural Products, Department of Chemistry, Center for Exact Sciences and Technology, Federal University of Maranhão, São Luís 65080-805, MA, Brazil.*

***** Correspondence: flaviaapmorais@hotmail.com (F.A.P.d.M.); cvnakamura@uem.br (C.V.N); Tel.: +55 (44) 3011-3680 (F.A.P.d.M. & C.V.N).

Supplementary Material 01. ^{1}H NMR spectra of F127-BT copolymer in D_2O at 25 °C and 300 MHz.

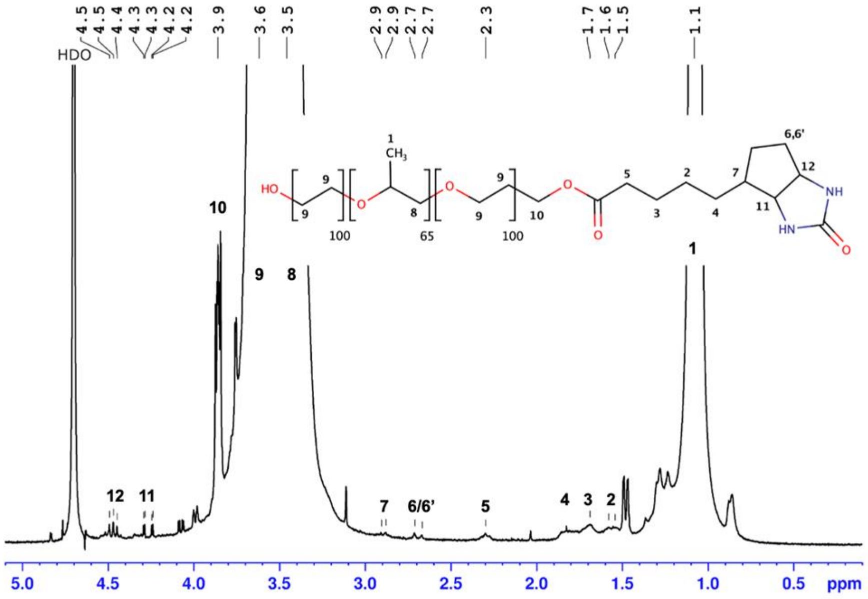

Supplementary Material 02. ^{1}H NMR spectra of F127-COOH copolymer in D_2O at 25 °C and 300 MHz.

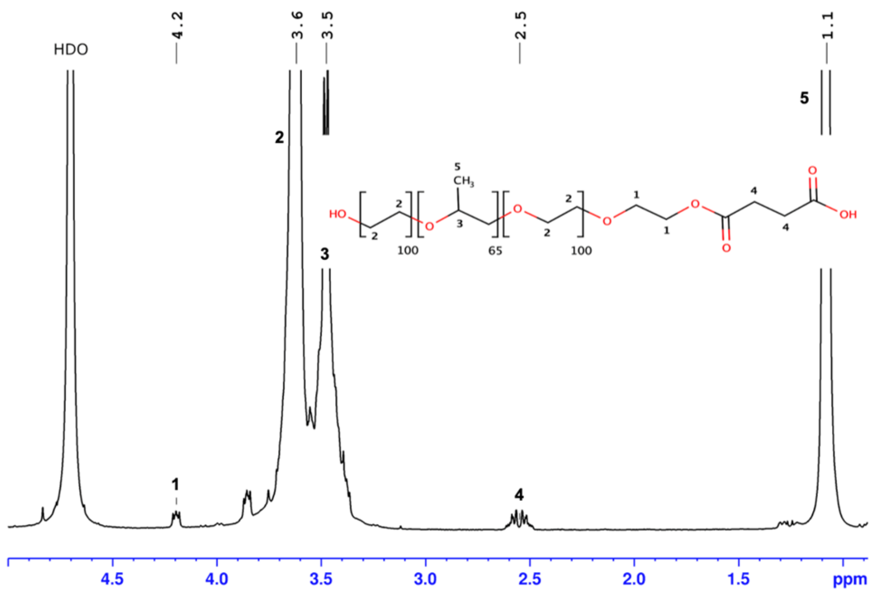

Supplementary Material 03. ^{1}H NMR spectra of F127 copolymer in D_2O at 25 ^{o}C and 300 MHz.

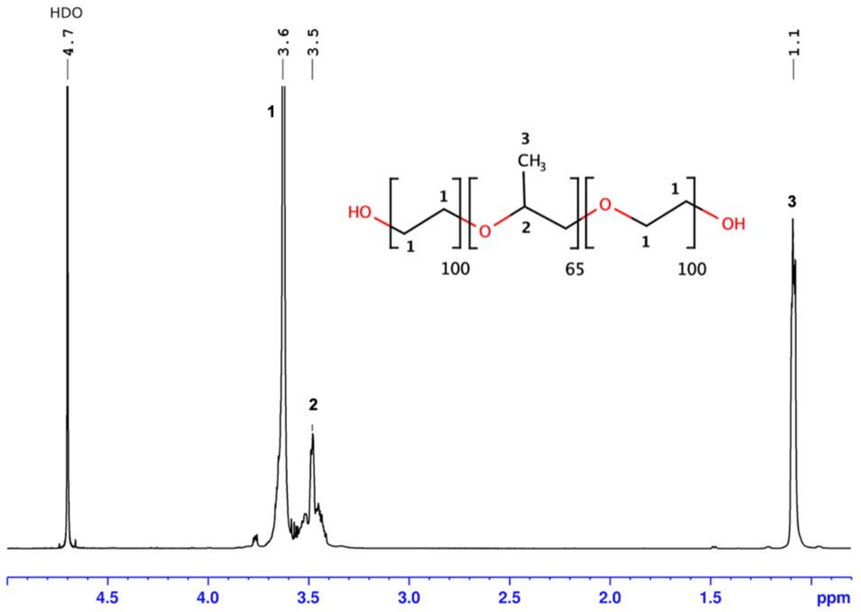

Supplementary Material 04. ^{1}H NMR spectra of F127-SN copolymer in D_2O at 25 ^{o}C and 300 MHz.

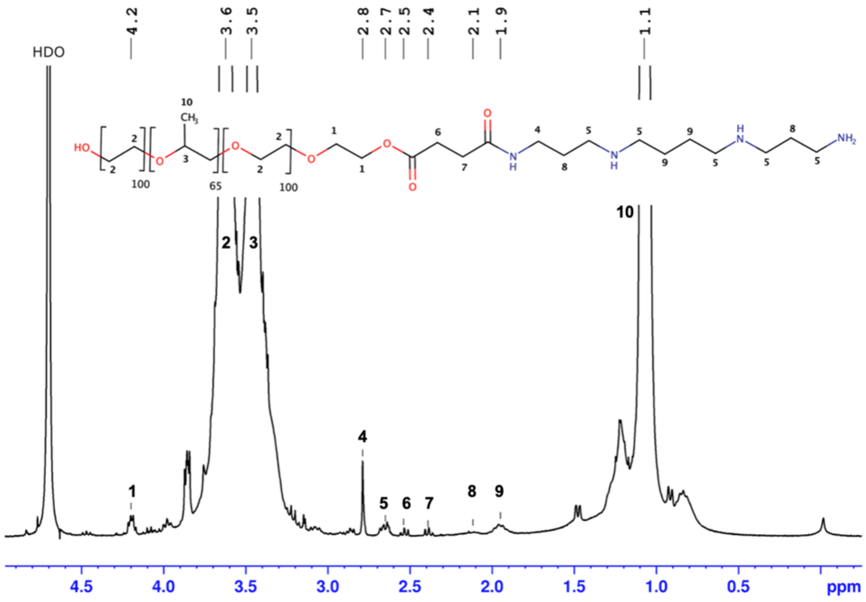

Supplementary Material 05. ^{1}H NMR spectrum of FA acquired in DMSO-d5 at 300 MHZ and 25 °C.

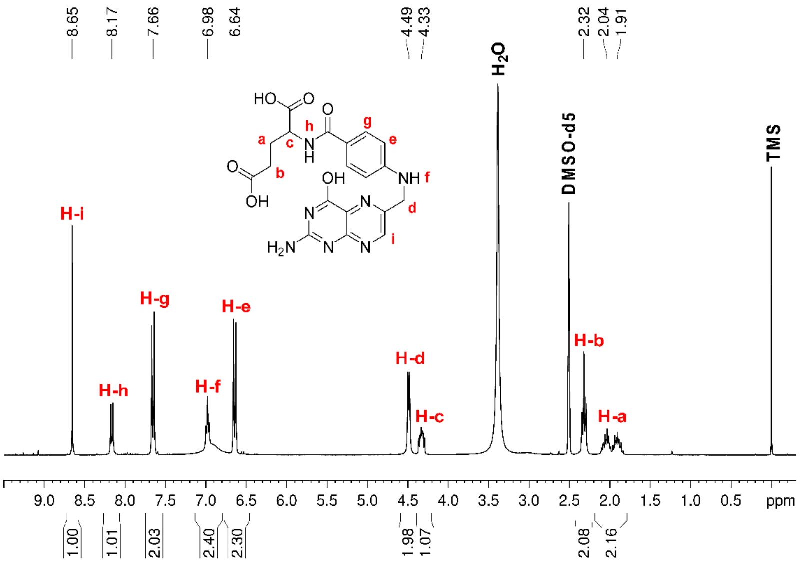

Supplementary Material 06. ^1H NMR spectra of F127-FA copolymer in D_2O at 25 ^oC and 300 MHz.

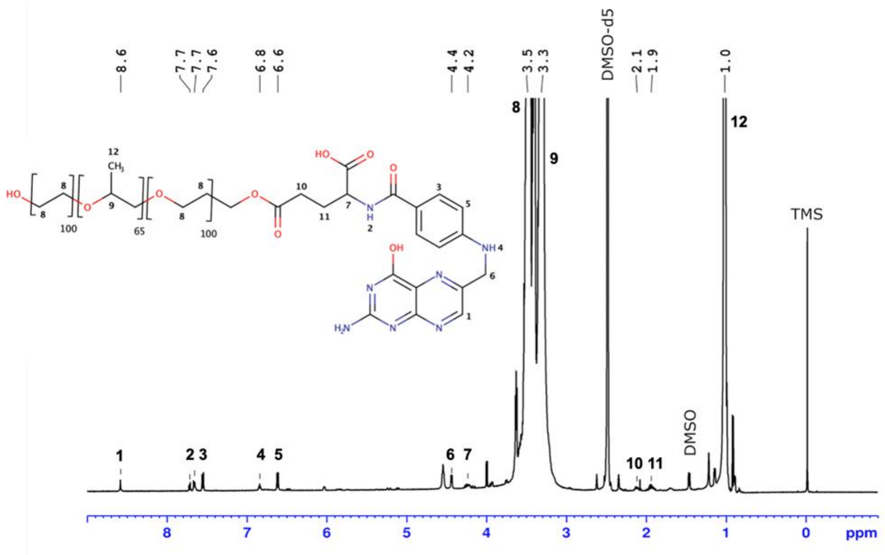

Supplement: Supplementary file 1 [file polymers-15-00179-s001.zip › polymers-2098469-supplementary.pdf]
